# Correlation between Cellular Structure Morphology and Anisotropic Yield in Additively Manufactured Stainless Steel 316L

**DOI:** 10.3390/ma16041666

**Published:** 2023-02-16

**Authors:** Dae Woong Kim, Soo Bin Han, Yoon Sun Lee, Dong Yong Park, Ho-Jin Lee, Sung Hyuk Park, Hyejin Song

**Affiliations:** 1 Analysis & Assessment Group, Research Institute of Industrial Science & Technology, Pohang 37673, Republic of Korea; 2 Smart Manufacturing Technology R&D Group, Korea Institute of Industrial Technology, Daegu 72994, Republic of Korea; 3 School of Materials Science and Engineering, Kyungpook National University, Daegu 41566, Republic of Korea

**Keywords:** additive manufacturing, stainless steel, cellular structure, Taylor factor, Anisotropy

## Abstract

Additively manufactured austenitic stainless steel 316L is composed of a cellular structure, which has a directionality, and is observed with a different morphology depending on the observation direction. The cellular structure morphology that appears with a high probability in grains with a specific grain orientation is determined. Taylor factor, which is calculated by considering grain orientation, is related to cellular structure morphology due to the directional cellular structure in additively manufactured austenitic stainless steel 316L. The Taylor factor affects the mechanical properties. The yield strength of additively manufactured SUS316L can be explained by the correlation between cellular structure morphology, grain orientation, and Taylor factor.

## 1. Introduction

Additive manufacturing (AM) can form complex shapes that are difficult to make with conventional processes such as rolling, forging, and casting. In addition, in line with the next-generation manufacturing trend of mass customization and flexible production, it has attracted attention as a new manufacturing technology in various industries such as automobiles, aerospace, chemicals, and marine [1,2,3,4,5]. Additively manufactured products experience complicated thermal cycles because of the process in which metal powder is repeatedly melted and solidified by a high-power laser or electron beam [2,6,7,8]. The continuous deposition in the AM process, results in a complex solidification microstructure, which can overcome the strength-ductility trade-off observed in conventional metal materials, while exhibiting excellent mechanical properties [1,9,10].

AM methods can be broadly classified into powder bed fusion (PBF) and direct energy deposition (DED). In particular, the DED method forms a melt pool by irradiating a high-power laser beam on the metal surface and simultaneously supplying metal power [7,11]. Because it is similar to the conventional welding method, it can be implemented on the surface of existing products, and can therefore be used for repair work. In addition, it is possible to manufacture alloys in real time using several types of powders simultaneously [4,11,12]. As mentioned above, the metal material manufactured by the AM method exhibits a complex solidification microstructure, as rapid melting and cooling repeatedly occur [13,14,15]. In particular, in the case of austenitic stainless steel, alloying elements are segregated along the cell boundaries in a face-centered cubic (FCC) matrix, and is known to have a sub-micro cellular structure decorated with alloying elements and tangled dislocations [2,10,16,17].

This cellular structure is related to the excellent combination of strength and ductility of the materials manufactured using AM. Cellular structure boundaries decorated with alloying elements and tangled dislocations contribute to the improvement of strength in the Hall–Petch relationship by interfering with the dislocation movement [2,10,18]. In addition, the cellular structure boundary can act as a dislocation nucleation site, so that the cell size can influence the strain hardening of the material [16]. Although there are many previous studies on the effect of cellular structure on mechanical properties, few studies focusing on the effect of the cellular structure morphology on the mechanical properties have been conducted.

Cell structure grows along the preferred growth direction, especially in crystals with FCC structure, the preferred growth direction is the <001> direction [2,19]. The cellular structure morphology depends on the observation direction of cellular structure grown in the grain, and research on the relationship between orientation of grain and cellular structure morphology is insufficient. In addition, growth direction of cell structure is affected by crystallographic texture, and research on the correlation between cell structure and texture is needed.

In this study, the correlation between cellular structure morphology, grain orientation, and the Taylor factor was analyzed through the microstructure analysis of the planes perpendicular to the building direction (BD) and transverse direction (TD) in SUS316L fabricated using the DED method. The effect of microstructure, particularly cellular structure, grain orientation, and Taylor factor on the yield strength was also analyzed. The difference in mechanical properties according to cell structure morphology was correlated through hardness and the Taylor factor. The difference in compression properties of the BD and TD directions was analyzed through the volume fraction analysis of each cellular structure morphology.

## 2. Materials and Methods

**Figure 1:**
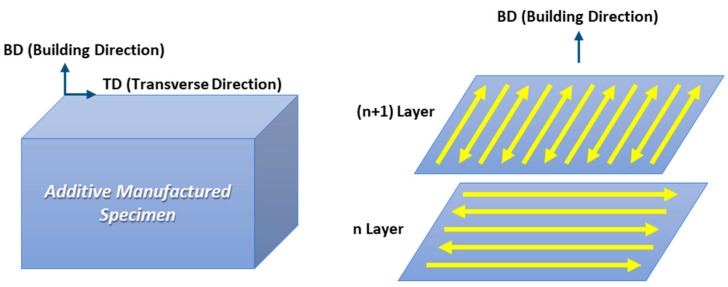
Schematic illustration showing the direction of the observation plane and scanning strategy in the additively manufactured specimen.

Austenitic stainless steel 316L powder was commercially produced and the average size of the power was 110 μm. The chemical composition (Fe-18.4Cr-12.2Ni-3.07Mo-0.02C (wt.%)) of the powder was measured by X-ray florescence (XRF, SU8020, Hitachi). The specimen was additively manufactured with a rectangular parallelepiped (dimensions: 20 × 20 × 20 mm) using a DED machine (model: MX-3, InssTek), with fixed parameters of a laser power of 400 W, scan speed of 0.85 m/min, powder feed rate of 3 g/min, hatching space of 0.5 mm, and layer thickness of 0.25 mm. The scan strategy is depicted in Figure 1, which represents a cross-snake hatching method that rotates the hatching direction by 90° after stacking each layer.

As depicted in Figure 1, the microstructures were analyzed in planes perpendicular to the transverse direction (TD) and building direction (BD), respectively. Additively manufactured stainless steel specimens were polished and etched in a solution of glycerol (45 mL), hydrochloric acid (30 mL), and nitric acid (30 mL), and the microstructures on the BD and TD planes of the specimens were observed using optical microscopy (OM) and field-emission scanning electron microscopy (FE-SEM, model: JSM-7900F, JEOL). The orientation analysis and the Taylor factor of the specimens were analyzed using electron back-scatter diffraction (EBSD) analysis (OIM Data Collection 5 software, step size; 0.8 μm). Specimens for EBSD were prepared by mechanical polishing using a 0.04 μm colloidal solution.

Cylindrical type specimens (diameter: 5π, height: 10 mm) were compressed parallel to transverse direction and building direction, respectively, using a universal testing machine (model: 5988, INSTRON) at room temperature with a strain rate of 10^−3^ s^−1^. All compression tests were performed three times to increase the reliability of the data. Vickers hardness tests for each cellular structure conducted 10 times at a constant load of 0.05 kgf (model: VH3300, BUEHLER).

## 3. Results and Discussion

**Figure 2:**
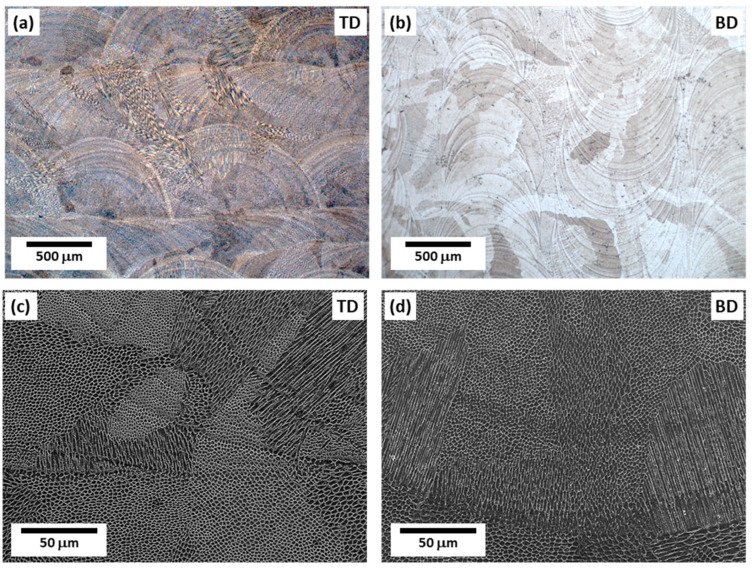
Microstructures of the specimen: (**a**,**b**) OM and (**c**,**d**) SEM images of the TD and BD planes, showing melt pool boundaries and cellular structures.

**Figure 3:**
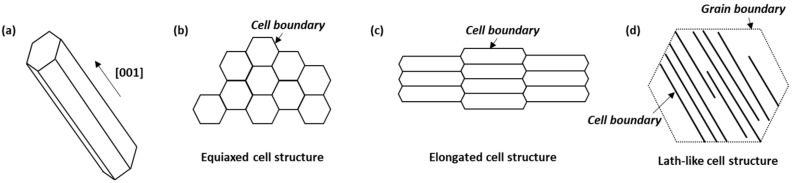
Schematic illustrations of (**a**) cell growth direction and (**b**–**d**) cellular structure types according to morphology.

The melt pool boundaries are clearly visible in the low-magnification OM images of the BD and TD planes (Figure 2a,b), and the SEM images depict how the morphology of the cellular structure differs based on the melt pool boundaries (Figure 2c,d). During AM, a complex microstructure is formed owing to rapid and repeated thermal cycles [13,14,15]. Cellular structures are formed through epitaxial growth along the preferred crystallographic orientation, which is close to the temperature gradient (G) direction form the molten pool boundaries. According to the solidification theory, the preferred crystallographic orientation is <001> in alloys with FCC crystals such as austenitic stainless steel 316L [2,19]. As shown in Figure 3, the cellular structures may have different morphologies depending on the direction of observation, due to epitaxial growth [2]. When the cell growth direction is parallel to the observation direction, equiaxed type appears, as depicted in Figure 3b. When the cell growth direction and the observation direction are close to 45 degrees, an elongated cell structure, which is intermediate between equiaxed and lath-like types, is observed (Figure 3c). When the cell growth direction and observation direction are vertical, a lath-like type is observed, as depicted in Figure 3d.

**Figure 4:**
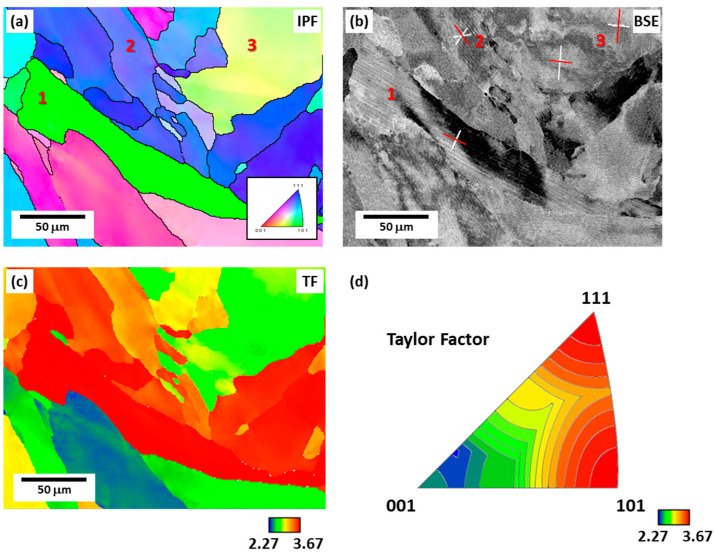
Microstructures of the TD plane: (**a**) EBSD inverse pole figure (IPF) map, (**b**) backscattered electron (BSE) image, (**c**) Taylor factor (TF) map, and (**d**) TF distribution according to grain orientation. Grain 1, denoted by the number 1, is composed of the lath-like cellular structure; grain 2 consists of both elongated type; and both elongated and lath-like cellular type are observed in grain 3.

Orientation analysis of the cellular structure morphology was performed based on the data obtained from EBSD (Figure 4). Figure 4a,b depicts the SEM backscattered electron (BSE) image in the same region as the EBSD inverse pole figure (IPF) image, corresponding to the TD plane of the specimen. Along the cellular structure boundaries, solute atoms are segregated during the solidification process due to the difference in solubility between solid and liquid, and a difference in contrast appears in the BSE image, which makes it possible to distinguish the cellular structure boundaries, thereby revealing the cellular structure morphology [2]. In Figure 4a,b, the grain composed of the cellular structure observed to be of lath-like type is grain 1; the grain observed as elongated type is grain 2; and the grain that appears as both elongated and lath-like cellular types is grain 3. The <001> plane traces of each grain are shown in the BSE image (Figure 4b). Here, grain 1 is identified as a grain composed of a typical lath-like cellular structure in which a parallel lath appears. Consequently, the lath direction is observed to coincide with one of the cell growth directions by comparing it with the red plane trace in the grain. Grain 2 is identified as cellular structure with an elongated shape, and the elongated direction coincides with the <001> direction. In the case of grain 3, elongated and lath-like types appear simultaneously. Because the growth of on grain is possible in each of the [001], [010], and [100] directions, up to three types of cellular structure morphologies can be observed. Therefore, two types of cellular structures can be observed simultaneously in grain 3, where the elongated directions are also well matched with <001> (Figure 4b), which is the direction of cell growth.

Because cellular structures grow along the <001> direction, the probability of determining each type of cellular structure varies depending on the grain orientation. The equiaxed type is more likely to be observed closer to the <001> oriented grain on the IPF, which coincides with the cell growth direction. There are three <001> directions, and in the <001> oriented grain, the probability of finding the equiaxed and lath-like types is high. Because the lath-like type must be perpendicular to the <001> direction, it is more likely to be found between the <001> oriented and <101> oriented grains on the IPF. Table 1 shows the number of cases of each type found according to the grain orientation in the IPF map. The closer to the <001> oriented grain, the higher the probability of equiaxed and lath-like cellular types grains; the closer to the <111> oriented grain, the higher the probability of elongated type grains; the closer to the <101> oriented grain, the higher the probability of finding elongated and lath-like cellular structures grains.

**Figure 5:**
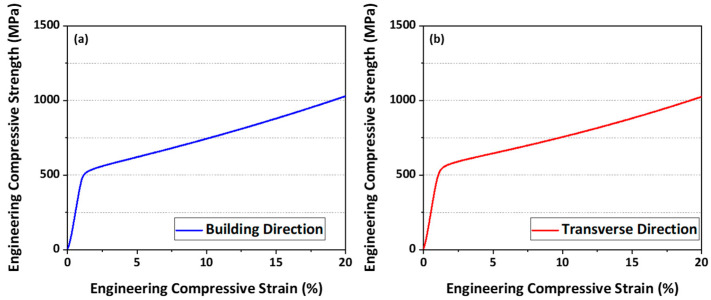
Room temperature engineering stress-strain curves obtained from the quasi-static compression tests in (**a**) building direction and (**b**) transverse direction.

Figure 5 shows the room temperature engineering compressive stress-strain curves obtained from quasi-static compression tests in two directions, BD and TD, and the average and standard deviation values of yield strength in the two directions are presented in Table 2. The average yield strengths of BD and TD are 520 MPa and 542 MPa and standard deviation of BD and TD are 2.5 MPa and 6.2 MPa, respectively. In other words, the yield strength of TD direction is 22 MPa higher than in the BD direction.

The Taylor factor, which was calculated by considering grain orientation, was analyzed form the EBSD data to explain the yield strength of the additively manufactured SUS316L specimen in relation to the microstructure, particularly the cellular structure morphology. The Taylor factor is a parameter defined according to the relationship among the direction of mechanical property evaluation, slip plane, and slip direction, and is closely related to the yield strength [20]. The yield strength is generally expressed by the Hall–Petch equation as follows [21]:

σ = σ_0_ + MαGb^1/2^, (1)

where σ and σ_0_ are the applied stress and friction stress; M is the Taylor factor; α is a constant between zero and unity; G is the shear modulus; b and ρ are Burgers vector and dislocation density. Consequently, the yield strength is observed to be proportional to the Taylor factor.

Figure 4c depicts a map showing the resulting Taylor factor distribution when deformation is applied along the BD in the same regions in Figure 4a,b. The slip plane and slip direction were set to (111)<110>, which is the main slip system of FCC materials. Figure 4d shows the TF distribution map, which shows the correlation between Figure 4a,c, calculated through EBSD when deformed in the BD direction. In Figure 4d, the <111> oriented grain and <011> oriented grain have a high Taylor factor, whereas the <001> oriented grain have a relatively low Taylor factor [22]. Thus, the close relationship between the grain orientation and Taylor factor is highlighted.

**Figure 6:**
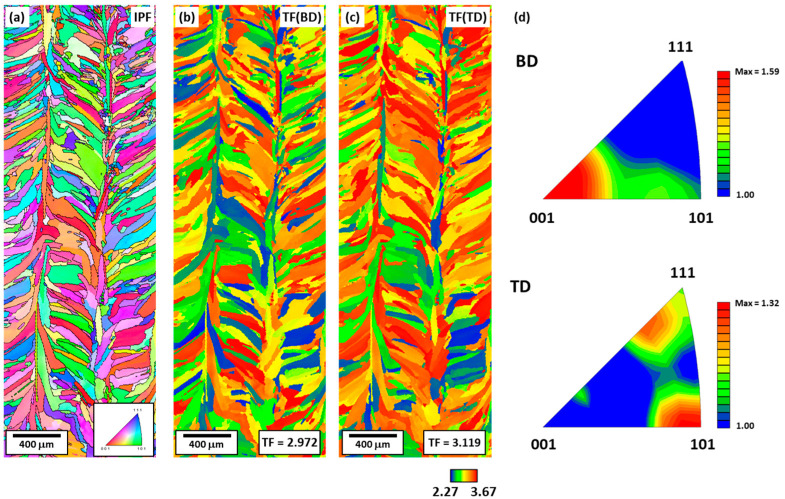
Microstructures of the BD plane: (**a**) EBSD IPF map; TF map under deformation parallel to (**b**) BD and (**c**) TD; and (**d**) IPFs along BD and TD showing orientation distribution.

Figure 6a shows the IPF map of the BD plane, and Figure 6b,c shows the Taylor factor map when deformation is applied along the BD and TD, respectively, along with the Taylor factor values. When evaluating the mechanical properties in the BD and TD, the Taylor factor values were calculated to be 2.972 and 3.119, respectively, and there was a higher Taylor factor value along the TD. As can be seen from Equation (1), this is consistent with the compression test result, in which the yield strength in the TD is higher. Figure 6d shows the distribution of the grain orientation parallel to the BD and TD. The BD//<001>, TD//<111>, and TD//<101> textures are predominant. From Figure 4d, it can also be seen that the Taylor factor is related to the grain orientation. In the BD, the <001> texture with a low Taylor factor is dominant; thus, the average Taylor factor value is low. In contrast, in the TD, the average Taylor factor value is high because of the <101> and <111> textures. In summary, the TD//<101> and TD//<111> textures are developed along the TD, resulting in a high Taylor factor and consequently, a higher yield strength than that along the BD.

Hardness is also related to the Taylor factor, and the higher the Taylor factor value, the higher the hardness [23,24,25]. Table 3 presents the hardness values according to the type defined by the cellular structure morphology. The hardness of equiaxed, elongated, and lath-like cellular structures were 218 HV, 244 HV, and 226 HV, respectively, and the standard deviations were 6.6 HV, 7.3 HV, and 14.5 HV, respectively. Therefore, the hardness was the highest in the elongated cellular structure and the lowest in the equiaxed cellular structure. In addition, the standard deviation was the highest in the lath-like cellular structure. The elongated cellular structure is more easily observed when approaching the <111> oriented grain, and additionally, the closer it gets to the <111> oriented grain, the higher the Taylor factor value; thus, the hardness value is also increased. Conversely, because equiaxed type is mainly found close to the <001> oriented grain, its Taylor factor value and hardness are relatively low. In the case of lath-like type, which is observed between the <001> and <101> oriented grains, there is a higher probability of it being observed in the <001> oriented grain; therefore, it has a low hardness with a large standard deviation of hardness.

**Figure 7:**
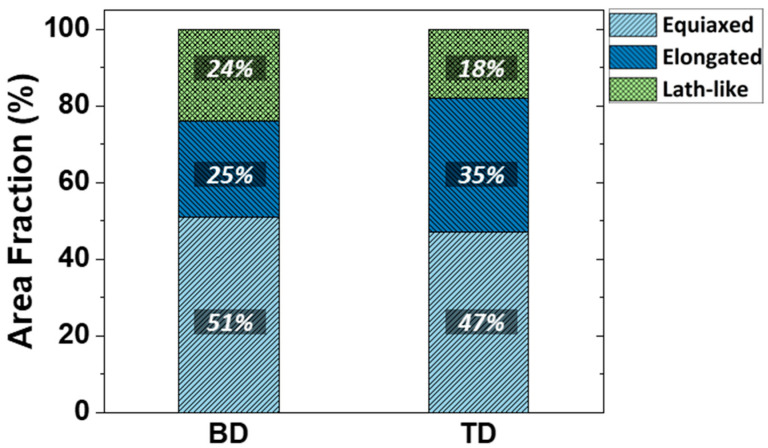
Area fraction of each cellular structure morphology in BD and TD planes.

The area fraction of each cellular structure morphology was measured from 50 SEM micrographs of the TD and BD plane using an image analyzer, respectively (Figure 7). In the TD planes, the fraction increases in the order of lath-like (18%), elongated (35%), and equiaxed (47%) whereas in the BD planes, the fraction increases in order of lath-like (24%), elongated (25%) and equiaxed (51%). In both planes, the cellular structure area fraction tends to be similar, but the area fraction values are different. TD plane has high area fraction of elongated type with a high Taylor factor and hardness, whereas BD plane has high area fraction of equiaxed type with a low Taylor factor and hardness. This is consistent with the tendency that the yield strength in the TD direction is higher than that in the BD direction.

In conclusion, because epitaxial growth with the directionality of the cellular structure occurs in the additively manufactured specimen, the cellular structure morphology varies depending on the angle between the observation and cell growth directions. Therefore, the cellular structure morphology is determined, which can have a high probability of appearance in grains with a specific grain orientation, and because the grain orientation can affect the Taylor factor, the morphology of the cellular structure affects the mechanical properties of the specimen, such as yield strength and hardness. In other words, in this study, it is confirmed that the elongated cellular structure morphology has a high probability of being found in grains with high Taylor factor, so the elongated cellular structure morphology has a higher hardness compared to other morphologies. As a result, the yield strength of the TD direction is higher because the TD plane has a higher elongated cellular structure fraction than the BD direction. Thus, yield strength of austenitic stainless steel additively manufactured can be predicted through cellular structure morphology and it can be helpful in designing 3D printed structures.

## 4. Conclusions

In this study, the yield strength of austenitic stainless steel additively manufactured using the DED method was explained in relation to the morphology and orientation of its cellular structure. The cellular structure appearing during AM underwent epitaxial growth along the <100> direction, resulting in equiaxed, elongated, and lath-like types, depending on the observation direction. In the specimen, the BD//<001> texture, in which the lath-like type was mainly observed along the BD, and the TD//<111> and <TD>//<101> textures, where the elongated type was mainly observed along the TD, were found. The values of the Taylor factor affecting the mechanical properties differed depending on the orientation. The equiaxed type had a low Taylor factor value; the elongated type had a high Taylor factor value; and the lath-like type had low or high Taylor factor values depending on the observation direction. Therefore, in the TD, the yield strength was higher than that in the BD owing to the microstructure having a high Taylor factor. From these research results, the possibility of application to predicting yield from the fraction or cellular structure morphology was confirmed.

## Figures and Tables

**Table 1:** Type classification according to grain orientation and cell growth direction.

Grain Orientation	Cell Growth Direction	Angle between Grain Orientation and Cell Growth Direction	Cellular Structure Morphology
(111)	[100]	54.7°	Elongated
	[010]	54.7°	Elongated
	[001]	54.7°	Elongated
(110)	[100]	45.0°	Elongated
	[010]	45.0°	Elongated
	[001]	90.0°	Lath-like
(100)	[100]	0°	Equiaxed
	[010]	90.0°	Lath-like
	[001]	90.0°	Lath-like

**Table 2:** Quasi-static compression test results of each direction.

Direction	Yield Strength (MPa)	
	Average (MPa)	Standard Deviation
BD	520	2.5
TD	542	6.2

**Table 3:** Vickers hardness according to cellular structure morphology.

Specimen	Cellular Structure Morphology	Average	Standard Deviation
SUS316L	Equiaxed Elongated Lath-like	218 HV 244 HV 226 HV	6.6 7.3 14.5

## Data Availability

The data presented in this study are available upon request from the corresponding author. The data are not publicly available due to confidentiality.
